# An Isolated Radiographic Finding of Spontaneous Vertebral Osteomyelitis

**DOI:** 10.7759/cureus.24646

**Published:** 2022-05-01

**Authors:** Collin Tacy, Veshesh Patel, Jorge Perez

**Affiliations:** 1 Osteopathic Medicine, Nova Southeastern University Dr. Kiran C. Patel College of Osteopathic Medicine, Fort Lauderdale, USA; 2 Internal Medicine, Brandon Regional Hospital, Brandon, USA

**Keywords:** discitis, empiric treatment, inflammatory markers, magnetic resonance imaging, vertebral osteomyelitis

## Abstract

Vertebral osteomyelitis (VO) is an infection of the vertebral body, most often arising secondary to hematogenous spread or contiguous spread from local soft tissue infection. Establishing a diagnosis of VO requires a high index of suspicion as patients often present with nonspecific symptoms such as pain of the affected vertebral segments along with leukocytosis and elevated inflammatory markers. Magnetic resonance imaging (MRI) has high sensitivity and specificity for detecting VO, even in the early phases of infection. Diagnosis is generally confirmed with blood cultures or vertebral biopsy demonstrating a culprit organism and treatment is tailored to the identified organism. However, some patients may have culture-negative VO that still necessitates antimicrobial treatment. Imaging alone may be an acceptable form of diagnosis that can allow for prompt empiric antibiotic therapy, reducing the need for invasive diagnostic measures. We present a case of a 46-year-old male with a past medical history of type 2 diabetes mellitus, hyperlipidemia, and prior transient ischemic attack (TIA). The patient presented with signs and symptoms of another TIA as well as new-onset neck and upper back pain. MRI in the neurologic workup demonstrated findings consistent with osteomyelitis of the C5 and C6 cervical vertebrae. Previous imaging showed no evidence of vertebral dysfunction. This patient presented with new-onset VO in the absence of systemic symptoms or elevation of inflammatory markers and no identified source of infection. Based on imaging and clinical presentation, empiric antibiotic treatment was initiated resulting in clinical improvement and resolution of VO on imaging. This case demonstrates an atypical presentation of VO and describes the benefit of MRI in recognizing infection in the absence of concurrent typical findings, which allowed for the initiation of empiric therapy.

## Introduction

Vertebral osteomyelitis (VO) is an infection of the vertebral body, most often arising from hematogenous spread or contiguous spread from local soft-tissue structures [1]. Establishing a diagnosis of VO requires a high index of suspicion when patients present with nonspecific clinical findings such as focal pain and tenderness of the affected vertebral segments. This is generally accompanied by elevated inflammatory markers such as erythrocyte sedimentation rate (ESR) and C-reactive protein (CRP), with ESR being positive in more than 90% of patients with VO [2]. Some cases present with other findings such as fever and leukocytosis, but these findings are less sensitive and specific to VO. When VO is suspected, magnetic resonance imaging (MRI) is the gold standard for diagnosis followed by a CT-guided biopsy of the identified lesion to isolate the offending pathogen. MRI has a high sensitivity, specificity, and accuracy for detecting VO and discitis, being reported as 96%, 94%, and 92%, respectively [2]. Antimicrobial treatment is often initiated when a diagnosis is established from biopsy results to employ appropriate therapy. However, some patients may have blood culture- and biopsy culture-negative VO that still necessitates antimicrobial treatment. This case details a patient with symptoms and radiographic findings indicative of VO identified on MRI. This patient was found to have no elevation of inflammatory markers, normal leukocyte count, and negative blood cultures with no identified source of infection.

## Case presentation

We present a case of a 46-year-old male with a past medical history of type 2 diabetes mellitus, hyperlipidemia, and prior transient ischemic attack (TIA). The patient presented to the emergency department with complaints of palpitations, chest pain, left arm and left leg weakness, and left-sided facial numbness. He also reported that he recently started having pain in his neck and upper back with radiation to the occipital region. An electrocardiogram (EKG), chest X-ray, and CT of the head and neck were completed as an initial stroke and cardiac workup, demonstrating no acute pathologic findings on admission.

**Figure 1:**
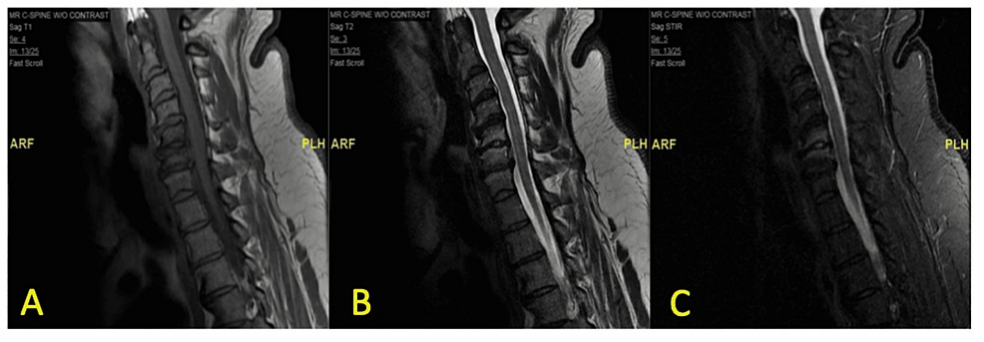
MRI of the cervical spine Sagittal view of the cervical spine demonstrates hypointense T1 (A) and hyperintense T2 (B) signals in the vertebral bodies of C5 and C6 as well as short tau inversion recovery (STIR) signal/postcontrast enhancement (C) in the inferior half of the C5 vertebral body and the superior half of the C6 vertebral body, consistent with osteomyelitis and discitis.

**Figure 2:**
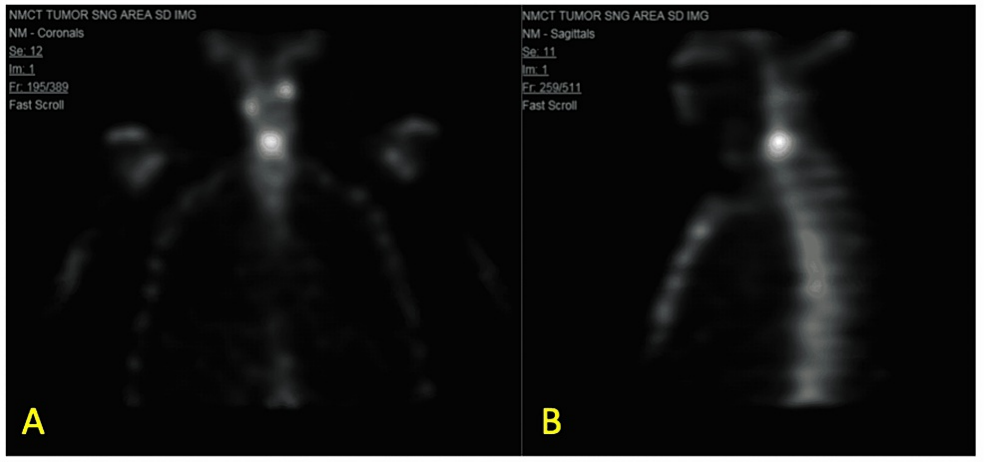
Nuclear medicine triple-phase bone scan Coronal view (A) and sagittal view (B) bone scan demonstrated increased radiotracer uptake in the vertebral bodies of the lower cervical region reflecting inflammatory changes.

**Figure 3:**
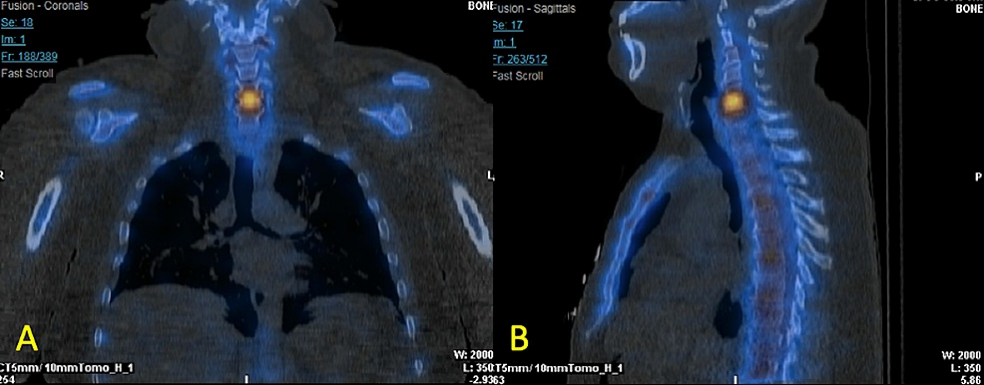
Single-photon emission computed tomography (SPECT) Coronal view (A) and sagittal view (B) SPECT demonstrated increased radiotracer uptake in the inferior half of the C5 vertebral body and the superior half of the C6 vertebral body and endplates, as well as the C5-C6 disc space, consistent with findings seen on MRI.

Further neurologic evaluation with MRI of the head and neck revealed findings consistent with osteomyelitis of the C5 and C6 cervical vertebrae and discitis of the C5-C6 intervertebral disc, as shown in Figure 1. Imaging findings were however inconsistent with the patient’s neurological complaints and deficits, likely reflecting separate etiologies. Follow-up imaging was performed with a nuclear medicine triple-phase bone scan with single-photon emission computed tomography (SPECT). Nuclear medicine bone scan demonstrated focal uptake of radiotracer in the lower cervical region, as shown in Figure 2. This was consistent with the focal uptake seen in the C5 and C6 cervical vertebral segments on SPECT, as shown in Figure 3. These findings were correlated with MRI findings and represented a diagnosis of VO and discitis.

**Figure 4:**
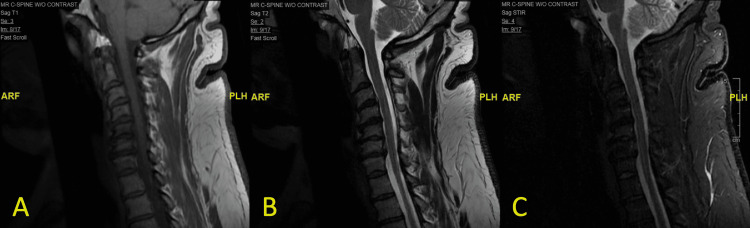
Repeat MRI of the cervical spine following empiric treatment Sagittal view of the cervical spine demonstrates moderate loss of disc space of the C5-C6 and C6-C7 intervertebral discs, unchanged from previous imaging. There is no vertebral osteomyelitis or discitis identified in the cervical spine on T1 (A), T2 (B), or short tau inversion recovery (STIR) (C) imaging.

The patient was offered further diagnostic options including a CT-guided biopsy of the C5 and C6 vertebral segments with the possible need for surgical fusion of C5 and C6 given the collapsed nature of the vertebral bodies and intervertebral disc. With the patient desiring a non-invasive diagnostic and treatment course, he was started on intravenous (IV) empiric antibiotic treatment for six weeks, consisting of daptomycin 800 milligrams (mg) and ceftriaxone two grams (g) daily, leading to significant improvement of his focal spinal tenderness. Repeat MRI of the cervical spine was performed 12 weeks after the initiation of IV antibiotic treatment and demonstrated radiographic improvement, with no VO identified, as shown in Figure 4.

## Discussion

VO is an infection of the vertebral body that often presents insidiously with nonspecific symptoms and highly variable clinical findings, necessitating diagnostic confirmation with imaging and biopsy [3]. The presented case demonstrates a case of VO and discitis that presented with atypical clinical findings and was identified on MRI, nuclear medicine bone scan, and SPECT.

Pathogenesis of vertebral osteomyelitis

Hematogenous dissemination is the most common route of infection in VO likely due to the rich blood supply of the vertebral bodies. The intervertebral discs, however, are largely avascular, receiving most of their nutrients from passive diffusion from the vertebral endplates [4]. This is consistent with the nidus of the infection being the vertebral bodies and endplates with subsequent spread to the disc space and adjacent vertebrae [5].

The greatest predisposing factors for the development of VO include diabetes mellitus, immunosuppression, malignancy, long-term corticosteroid use, spinal instrumentation, and preceding bacteremia. Many of these risk factors make it difficult for the body to eradicate the infection, which is most often due to a single pathogen [6].

Diagnostic methods and impact on treatment

A diagnosis of VO requires a high index of suspicion that starts with recognizing the most common clinical manifestation, i.e., pain. Localized pain of the affected vertebral segments is usually insidious in onset and experienced with vertebral motion or palpation of the affected region. Patients may present with fever and leukocytosis, which are unreliable findings, as they are neither sensitive nor specific for VO. The most common laboratory findings in patients with VO include elevations in inflammatory markers such as ESR and CRP, which are seen in greater than 80% of patients [1]. However, serum inflammatory markers have been shown to vary based on the responsible infecting organism. A more robust elevation is seen with *Staphylococcus aureus* and antibiotic-resistant organisms as compared to the minimal elevation seen with blood culture- and biopsy culture-negative cases [7]. Given this, the variation in inflammatory marker elevation may have a correlation to the positive versus negative cultures. The best diagnostic test to evaluate for the presence of VO and its sequelae is an MRI that would demonstrate decreased intensity on T1-weighted imaging and increased intensity on T2-weighted imaging of the vertebral bodies and intervertebral disc. Although these findings may also be seen with degenerative changes in the spine (Modic type I changes), the presence of vertebral endplate involvement and enhancement on short tau inversion recovery (STIR) and postcontrast imaging is more consistent with VO [5].

Alternative imaging techniques with comparable sensitivity to MRI for the detection of VO include positron emission tomography (PET) and SPECT [6]. Due to the limited specificity of PET and SPECT, these studies are more reliable when used in conjunction with MRI to gather a more definitive picture of VO.

When VO is identified on imaging in the absence of complications warranting emergent surgical intervention, such as the neurological deficit, abscess, or spinal cord compression, the next step in management generally involves biopsy of the affected segments to guide antimicrobial therapy [1]. A biopsy is most often performed as CT-guided or fluoroscopic-guided approach; however, diagnostic yield may be as low as 53%. Open biopsy tends to have a higher positive yield (up to 91%) but at the cost of increased morbidity [5]. Some have even suggested that biopsy should only be performed in cases of poor response to initial therapy or when surgical intervention is warranted, as empiric regimens are often kept the same regardless of culture positivity [8].

In general, treatment should be directed toward the isolated organism, if identified. When clinical suspicion remains high in the setting of negative cultures, an empiric regimen should be initiated for coverage of staphylococci, streptococci, and gram-negative bacilli. This consists of vancomycin plus cefotaxime, ceftazidime, ceftriaxone, cefepime, or ciprofloxacin for a minimum of six weeks [1].

## Conclusions

This case demonstrates an atypical presentation of VO with no acute signs of infection, such as elevation in inflammatory markers, fever, or leukocytosis. The diagnosis was discovered as an isolated finding on MRI, which was further supported by findings on SPECT and nuclear medicine imaging. Despite the inability to obtain a biopsy of the lesion, the patient’s infection was amenable to empiric antibiotic treatment. This case illustrates the importance of initiating treatment for patients in whom the diagnosis of VO can be established on imaging, as the use of empiric antibiotic therapy may be helpful in reducing patient morbidity associated with invasive biopsy.
